# Postoperative Anisocoria-need not be Concerned Always

**DOI:** 10.4274/TJAR.2022.221013

**Published:** 2023-06-16

**Authors:** Ashutosh Kaushal, Roshan Andleeb, Priyanka Gupta, Praveen Talawar

**Affiliations:** 1 Department of Anaesthesiology, All India Institute of Medical Sciences, Bhopal, India; 2 Department of Anaesthesiology, All India Institute of Medical Sciences, Rishikesh, India

**Keywords:** Anisocoria, benign episodic unilateral mydriasis, migraine, neuroanaesthesia, prone position

**Dear Editor,**

Anisocoria in the postoperative period may indicate life-threatening conditions, and the possible causes are intracranial pathologies, Horner syndrome, acute angle closure glaucoma, ocular injury, or pharmacological blockade.^1,2^ We report a unique case of postoperative anisocoria in a patient who underwent cervical spine surgery in a prone position with the patient’s head secured on a head clamp.

A 30-year-old female patient underwent posterior cervical spine surgery because of a C5-C6 fracture following a road traffic accident under standard general anaesthesia in a prone position with her head secured on a Mayfield 3-pronged head clamp. In preoperative anaesthesia check-up, she was ASA I, had a stable vitals, 15/15 Glasgow coma score (GCS), normal bilateral size reacting pupils, and no neurological deficit. The patient had a history of migraine for five years. The surgery continued for three and a half hours. The intraoperative period was uneventful and there was 100-200 mL blood loss. Before extubation, the right-sided pupil was dilated (7 mm), sluggishly reacting to light, and the left pupil was 4 mm in size, normally reacting to light. Since the patient’s head was fixed on a Mayfield 3-pronged head clamp, non-contrast computed tomography (NCCT) was performed to rule out extradural haemorrhage, which was expected. The trachea was extubated as the patient was fully awake and moving all four limbs on command. During her stay in the post-anaesthesia care unit, the vitals and GCS remained normal, but anisocoria persisted. There was no associated headache, orbital pain, ptosis, facial anhidrosis, periorbital oedema, conjunctival chemosis, or lacrimation. Ophthalmology evaluation revealed 6/6 visual acuity in both eyes, normal intraocular pressure, and normal fundus examination. Thus, it was advised to wait for spontaneous recovery. The anisocoria gradually resolved more than one day, and the pupillary reaction also returned to normal. The patient and her relative were asked about any episode of anisocoria before, but they were unaware of it. She was followed up daily until discharge on the 10^th^ day and was uneventful.

Acute angle-closure glaucoma due to raised intraocular pressure may also cause unilateral dilation, but there are associated ocular pain, conjunctival hyperemia, or corneal edema.^3^ Horner syndrome also leads to anisocoria, but there is an associated triad of miosis, anhidrosis, and ptosis.^1,2^

Stroke, cerebral oedema, or intracranial hematoma could be the cause of anisocoria. An abnormal NCCT head and GCS deterioration are hard to miss in such scenario.^1^

Inability to maintain a neutral head position causing impaired venous return also leads to unilateral dilated pupil but concurrent exophthalmos due to venous congestion. The accidental direct pressure on the globe in a prone position may lead to postoperative anisocoria when the patients head is kept on a horseshoe headrest.^3^

In this case report, pressure-induced ocular injury could not be the reason for anisocoria because the head was secured on the Mayfield head frame. Pin site epidural hematoma was also overruled in view of normal NCCT head and full GCS. Pharmacological blockade can cause mydriasis without pain, ptosis, or diplopia, but such a dilation usually bilateral.

Adie’s pupil is a benign and idiopathic condition in which anisocoria can be precipitated by disruption of the sympathetic-parasympathetic balance.^1,2^ All these possible causes were ruled out in our case.

Benign episodic unilateral mydriasis (BEUM) is an isolated benign reason for intermittent pupil asymmetry, which may be associated with migraine. It can be present in migraine without aura or ophthalmoplegia.^4^ Functional exhaustion of parasympathetic fibers running within the III^rd^ cranial nerve, ischemia or oculomotor nerve demyelination caused by neuropeptides secreted at the level of the circle of Willis upon activation of the trigeminovascular system causing edema and inflammation may explain the reason for mydriasis in ophthalmoplegic migraine.^5^ These isolated benign episodic mydriasis have a benign neurological prognosis and do not necessitate further neurodiagnostic workup.

We assumed that it was a rare case of BEUM in the postoperative period in a female patient with a migraine history after excluding all possible causes of anisocoria. Migraine may be precipitated due to stress caused at the time of extubation. More research is needed to fully understand the underlying pathophysiology of BEUM in association with migraine.

Anisocoria during the perioperative period should be thoughtfully evaluated, as etiology ranges from serious life-threatening situations to benign local causes. Anaesthesiologists should be aware of this rare association of anisocoria with migraine in operative settings.
